# Diet and *PPARG2* Pro12Ala Polymorphism Interactions in Relation to Cancer Risk: A Systematic Review

**DOI:** 10.3390/nu13010261

**Published:** 2021-01-18

**Authors:** Lieu Tran, Gerd Bobe, Gayatri Arani, Yang Zhang, Zhenzhen Zhang, Jackilen Shannon, Yumie Takata

**Affiliations:** 1 College of Public Health and Human Sciences, School of Biological and Population Health Sciences, Oregon State University, Corvallis, OR 97331, USA; bichlieu.osu@gmail.com (L.T.); aranin@oregonstate.edu (G.A.); zhangya3@oregonstate.edu (Y.Z.); 2 Linus Pauling Institute, Oregon State University, Corvallis, OR 97331, USA; gerd.bobe@oregonstate.edu; 3 Knight Cancer Institute, Oregon Health and Science University, Portland, OR 97239-3098, USA; zhanzh@ohsu.edu (Z.Z.); shannoja@ohsu.edu (J.S.)

**Keywords:** peroxisome proliferator-activated receptor-γ2, *PPARG2* Pro12Ala polymorphism, diet, nutrients, cancer, diet-gene interactions

## Abstract

Peroxisome proliferator-activated receptor-γ2 gene Pro12Ala allele polymorphism (*PPARG2* Pro12Ala; rs1801282) has been linked to both cancer risk and dietary factors. We conducted the first systematic literature review of studies published before December 2020 using the PubMed database to summarize the current evidence on whether dietary factors for cancer may differ by individuals carrying C (common) and/or G (minor) alleles of the *PPARG2* Pro12Ala allele polymorphism. The inclusion criteria were observational studies that investigated the association between food or nutrient consumption and risk of incident cancer stratified by *PPARG2* Pro12Ala allele polymorphism. From 3815 identified abstracts, nine articles (18,268 participants and 4780 cancer cases) covering three cancer sites (i.e., colon/rectum, prostate, and breast) were included. CG/GG allele carriers were more impacted by dietary factors than CC allele carriers. High levels of protective factors (e.g., carotenoids and prudent dietary patterns) were associated with a lower cancer risk, and high levels of risk factors (e.g., alcohol and refined grains) were associated with a higher cancer risk. In contrast, both CG/GG and CC allele carriers were similarly impacted by dietary fats, well-known PPAR-γ agonists. These findings highlight the complex relation between *PPARG2* Pro12Ala allele polymorphism, dietary factors, and cancer risk, which warrant further investigation.

## 1. Introduction

Peroxisome proliferator-activated receptor-γ (PPARG or PPAR-γ) belongs to the nuclear hormone receptor superfamily, which induces target gene expression by binding as a heterodimer with retinoid X receptor alpha to PPAR-response element, the specific DNA motifs [1,2,3,4]. The three known splice variants of PPAR-γ include *PPARG1*, *PPARG2*, and *PPARG3*, with *PPARG2* being the longest and most bioactive [5,6]. Several diet-derived and synthetic small lipophilic compounds can bind to *PPARG* and modify its activity, either fully or partially promoting, or completely inhibiting it [1,2,7]. The most well-known PPAR-γ agonists are long- and very-long-chain fatty acids, and their derivatives and thiazolidinediones such as rosiglitazone and piaglitazone. They play a role in the PPAR-γ ’s most important metabolic function, which is to remove excess fatty acids and glucose from circulation through insulin signaling pathways [8]. PPAR-γ agonists, such as thiazolidinediones, are beneficial for type 2 diabetes management; however, thiazolidinediones fell out of favor because of severe side effects such as adipose hypertrophy, edema, and coronary dysfunction [9]. Thus, partial PPAR-γ agonists such as resveratrol, β-cryptoxanthine, isorhamnetin, and Gleevec have received increased attention for management of chronic diseases, including cancer [10].

PPAR-γ plays a role in adipocyte differentiation as well as carcinogenesis. Up-regulation of PPARG expression frequently occurs in many metabolic disorders [11] and cancers [12]. The *PPARG2* Pro12Ala polymorphism (rs1801282; chr3:12351626) is a coding and missense variant where a C (common) allele is replaced by a G (minor) allele, which leads to a substitution of proline (Pro) with alanine (Ala) in the 12th amino acid from the N-terminal end of *PPARG2*. This attenuates (−30%) PPARG2 activity and decreases the risk of type 2 diabetes and colorectal and breast cancers [13,14,15]. In contrast, the risk of obesity and gastric cancer are lower among C allele carriers [16,17], highlighting the complex involvement of PPAR-γ in carcinogenesis. This may be due to the fact that etiological and dietary factors differ by cancer site, and their associations may also differ by *PPARG2* Pro12Ala allele polymorphism. This notion is highlighted in previous studies of cardiometabolic diseases. For example, polyunsaturated fatty acid intake was inversely associated with risk of myocardial infarction among CC allele carriers, but not in G allele carriers (CG and GG) [18]. Total dietary fat intake had a statistically significant positive association with plasma high-density lipoprotein concentration among G allele carriers, but had a non-significant inverse association among CC allele carriers [19]. Furthermore, the fact that the number of common C alleles is associated with increased risk of chronic diseases such as type 2 diabetes [20] represents the public health impact. To the best of our knowledge, there has been no systematic literature review that examined whether the association of cancer risk with dietary factors differed by *PPARG2* Pro12Ala allele polymorphism, which is the objective of the current study.

## 2. Materials and Methods

### 2.1. Registration

**Figure 1:**
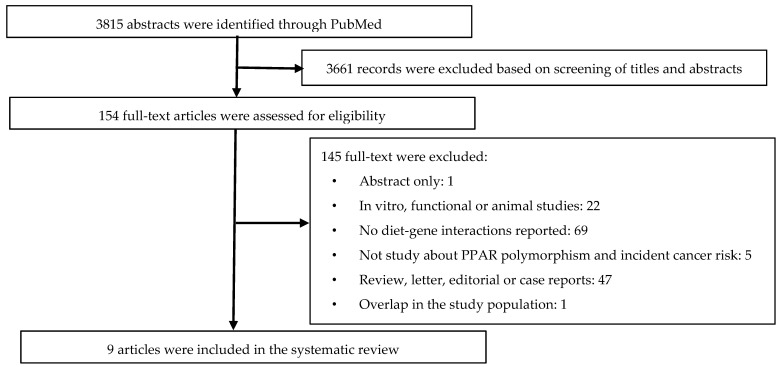
Flow chart of systematic literature review.

This systematic review was conducted according to the guideline of PRISMA (Preferred Reporting Items for Systematic Review and Meta-analysis) [21], and the protocol is registered with PROSPERO International Prospective Register of Systematic Reviews (CRD42020108352). Details of the systematic review and article selection steps are shown in Figure 1.

### 2.2. Search Strategy

This systematic review was conducted using the electronic database, PubMed, in December 2020 as the last search. In order to find eligible studies, the search terms listed in Supplementary Material were used. All titles and abstracts identified were screened for the inclusion of possibly eligible studies and exclusion of irrelevant studies. Three authors (L.T., G.A., and Y.T.) conducted this screening and review independently, and inconsistencies were discussed and brought to consensus. For studies with relevant titles and/or abstracts or studies that did not have enough information in the abstracts to make a decision, full-text articles were obtained. The full-text articles were evaluated for inclusion or exclusion.

### 2.3. Study Inclusion and Exclusion Criteria

The eligible studies needed to meet the following criteria: Human studies, written in English, and reporting the diet-gene (*PPARG2* Pro12Ala allele variant [rs1801282]) interaction in relation to incident cancer risk. Titles and abstracts, and/or full texts were excluded if they meet one of the following criteria: (1) Review, letter, editorial, commentary, or case reports; (2) in vitro, functional, or animal studies; (3) study not about *PPAR* polymorphism and incident cancer risk; (4) only abstract available; (5) no diet-gene interactions reported; or (6) overlap of the study population in another eligible original study or duplicated with individual studies in eligible meta-analysis.

### 2.4. Data Extraction

Data were extracted from each included study. Specific data extracted are: Last name of the first author; year of publication; study design; sample size (the number of cases/controls in case-control studies, or the number of total participants in other types of studies); study population characteristics (e.g., age, race, and sex); study location; cancer site; *PPARG2* Pro12Ala allele polymorphism; exposure or modifiable factors; interaction-related information such as risk estimates in the form of odds ratio (OR), relative risk, or incidence rate ratio (IRR), and 95% confidence interval (CI); and the statistical significance of the interactions. Two authors (L.T. and Y.T.) extracted data from each study independently, and inconsistencies were discussed and brought to consensus.

### 2.5. Risk of Bias Assessment

The risk of bias for each study was assessed by two authors (L.T. and Y.T.) independently using Newcastle–Ottawa scale for case-control and cohort studies [22]; any discrepancies were discussed and brought to a consensus. 

## 3. Results

Figure 1 shows the flow chart of results in the identification, screening, exclusion, and inclusion phases in the systematic review. First, as the identification phase, the search strategy on the PubMed database resulted in 3815 records. There were no duplicate abstracts among these records. Second, screening of titles and abstracts resulted in exclusion of 3661 records. Among the remaining 154 records, full-text articles were obtained and assessed for eligibility. Among those, 145 articles did not meet the inclusion criteria and were thus excluded. In total, our systematic review included nine articles, which included a total of 18,268 participants with 4780 cancer cases from eight studies (two articles are based on the same case-control study [23,24]). 

The eight studies included in our systematic review were conducted in a wide variety of countries; two were carried out in Denmark, and each of the other studies were conducted in the United States, Finland, Spain, India, Korea and Japan (Table 1). In terms of the study design, four studies were hospital-based case-control studies, three were nested case-control studies, and one was a population-based case-control study. A total of three cancer sites were investigated, including six studies on colorectal cancer, one on breast cancer, and one on prostate cancer. For the age of the participants, five studies reported the mean/median age in late fifties to early sixties, two studies reported age ranges between 20 and 79 [23,24,25], and one study did not report exact range or mean/median age [26]. In terms of sex of the participants, for six studies of colorectal cancer, the proportion of women ranged from 36% to 48%. Two studies of breast or prostate cancer were limited to postmenopausal women [27] or male smokers [28], respectively. All studies used a food frequency questionnaire to assess food and nutrient intakes. Dietary factors and cancer sites we identified are classified into two groups: (1) Alcohol (two nested case-control studies of colorectal and breast cancer); and (2) food and nutrient intakes (five colorectal cancer studies and one prostate cancer study). All studies had high quality with eight or nine score (good quality) (Table S1).

Alcohol and PPARG2 Pro12Ala allele variant: Two case-control studies nested within a Danish cohort [27,29] reported that *PPARG2* Pro12Ala modified the association between alcohol intake and cancer risk (Table 2). For breast cancer, alcohol intake increased the risk of breast cancer in CC allele carriers (IRR = 1.13 and 95% CI = 1.04–1.23), who had an overall higher risk than G allele carriers. In contrast, for colorectal cancer, alcohol intake was associated with an increased risk in G allele carriers (IRR = 1.22 and 95% CI = 1.07–1.39), who had a numerically overall higher colorectal cancer risk.

Diet and PPARG2 Pro12Ala allele variant: Six studies investigated whether *PPARG2* Pro12Ala polymorphism altered the association of nutrient and food consumption with cancer risk [23,24,25,26,28,30], of which five focused on colorectal cancer (Table 2, Table 3 and Table 4 and Table S2). In a US population-based case-control study [23], G allele carriers had a lower risk of colon cancer than CC allele carriers at the same high β-carotene and lutein intakes (Table 3). In a Spanish hospital-based case-control study [26], the inverse association with vitamin A intake was observed among all participants, which was stronger among G allele carriers than CC allele carriers at the low vitamin A intake. Moreover, *PPARG2* Pro12Ala polymorphism altered the association of dietary patterns and refined grain intake with cancer risk (Table 4); G allele carriers had a lower risk of colon cancer at high prudent dietary pattern scores and low refined grain intake than CC allele carriers [23]. In contrast, G allele carriers were more susceptible to high fried food intake (Table 2) and tended to be more susceptible to high egg and dairy product consumption (Table S1) than CC allele carriers [25,30]. The inverse association of colorectal cancer risk with fish intake was not altered by *PPARG2* Pro12Ala polymorphism (Table 2), as was the positive association with meat and fat intake (Table S2) [25,30,31]. 

## 4. Discussion

Through our systematic literature review, we identified eight case-control studies (18,268 participants and 4780 cancer cases) covering three continents (i.e., America, Asia, and Europe) and three cancer sites (i.e., colon/rectum, prostate, and breast) that examined the association between dietary factors and cancer risk among participants who differ in *PPARG2* Pro12Ala polymorphism. Overall, major findings from these studies were on: (1) Alcohol intake and breast or colorectal cancer risk; and (2) diet and colorectal cancer risk.

Dietary factors and *PPARG2* Pro12Ala polymorphism generally play a more important role in the etiology of colorectal cancer than other cancer sites [14,32,33,34,35]. PPARG expression levels in colorectal tissue are moderate to high [36,37,38]. Some fatty acids and their derivatives can bind to PPAR-γ and act as full agonists to promote cellular fatty acid uptake and lipogenesis, whereas carotenoids, lutein, and polyphenols can act as partial agonists and inhibit growth of colon cancer cell lines [1]. In three studies of colorectal cancer [23,29,30], high intake of cancer-protective factors—β-carotene, lutein and prudent dietary pattern score—had a stronger inverse association with risk of colorectal cancer among G allele carriers [23]. In contrast, high intake of cancer risk factors—refined grains and alcohol—had a stronger positive association with risk of colorectal cancer among G allele carriers [29,30]. Hence, G allele carriers may benefit more from dietary interventions than CC allele carriers.

Regarding prostate cancer, we found only one study, a nested case-control study among male smokers in Finland. This study investigated the interaction of dietary fat intake with *PPARG2* Pro12Ala variant and reported no statistically significant interaction [28]. There is a link between PPAR-γ and prostate cancer, as *PPARG1* acts as an oncogene and PPARG2 inhibits proliferation in prostate cancer cells [39]. Although some fatty acids (including long-chain and very long-chain unsaturated fatty acids and their derivatives) can bind to PPAR-γ as full agonists, limited evidence exists on dietary fat intake as a risk factor for prostate cancer, which might have led to the null finding [40]. Furthermore, a direct effect of PPAR-γ on prostate carcinogenesis is less likely, as PPARG levels are non-detectable in healthy prostate tissue [36,37]. Given that only one study reported the interaction between dietary factors and *PPARG2* Pro12Ala variant in prostate cancer, the interaction needs to be investigated in other study populations.

One study of breast cancer examined its association with alcohol consumption among postmenopausal women after stratifying by *PPARG2* Pro12Ala polymorphism. In general, CC allele carriers had an increased risk of breast cancer compared with G allele carriers [15]. PPAR-γ agonists, as well as PPAR-γ antagonists, can inhibit breast growth depending on type of breast cancer (e.g., estrogen receptor status) [41,42,43,44], which makes investigation of dietary factors and breast cancer challenging. In breast tissues from breast cancer patients, PPARG expression levels were lower in cancer than normal tissues and differed by cancer stage [44]. In a Danish nested case-control study, in comparison to G allele carriers, CC allele carriers had a higher risk of postmenopausal breast cancer with higher alcohol consumption. Their median alcohol consumption was less than moderate consumption (9 g/day) [27], which may be more of a concern for female CC allele carriers in regard to breast cancer risk. Given that only one study reported the diet-gene interaction for breast cancer, future studies need to replicate this finding.

Other than the three cancer sites (i.e., colon/rectum, prostate, and breast), we did not find any study of other cancer sites that investigated the interaction between diet and *PPARG2* Pro12Ala allele polymorphism. In the future, additional studies are warranted to elucidate the link between bioactive food components and cancer risk at sites with moderate to high PPARG expression such as the digestive tract (specifically gastric cancer) with high levels of bioactive food components [36,37]. Regarding dietary factors, more partial PPAR-γ agonists need to be explored, given that our systematic review found that associations between full PPAR-γ agonists and cancer risk did not differ by *PPARG2* Pro12Ala allele polymorphism, although limited studies were available. This may be due to side effects of full PPAR-γ agonists reported for type 2 diabetes, which might have nullified beneficial effects. Moreover, CC allele carriers were more susceptible to colorectal cancer and G allele carriers were more susceptible to gastric cancer [14,17], which requires further clarification.

This study has several strengths. To our knowledge, this is the first systematic review on diet-gene interactions of *PPARG2* Pro12Ala allele polymorphism in the association with cancer risk. We were able to include a large number of study participants in this review (18,268 participants). Moreover, we included previous studies conducted from a wide range of geographic locations, which facilitated to cover a wider range of dietary factors that could not have been achieved within one study population. This study also has limitations. First, we used only one database, PubMed, with potentially a limited number of studies available. Moreover, although all included studies had good quality based on the risk of bias assessment, they were not without potential bias nor were we able to fully control the quality of these studies. There was heterogeneity in reporting of interaction effects among the studies included in this review, which did not allow us to conduct a meta-analysis. Hence, we provided a comprehensive review with detailed descriptions. Future studies of diet-gene interactions need to consider more uniform reporting, such as by modeling low dietary intake and common allele groups as reference.

In conclusion, our systematic literature review found limited evidence on modifying effects of *PPARG2* Pro12Ala polymorphism on the association between dietary factors and cancer risk. For colorectal cancer, the most studied cancer site, risk factors (i.e., fried foods, alcohol, and refined grains) were more detrimental in individuals carrying the G allele, whereas high levels of protective factors (i.e., carotenoids and prudent dietary pattern score) were more beneficial in individuals carrying the G allele. In contrast, dietary fat and meat intakes were equally detrimental, and fish intake was equally protective in both *PPARG2* Pro12Ala genotypes, which need to be confirmed in future studies. More evidence is currently available on interactions of *PPARG2* Pro12Ala polymorphism with dietary factors other than well-known full PPAR-γ agonists, suggesting that partial PPAR-γ agonists or other novel bioactive compounds may be more relevant for the PPAR-γ’s involvement in carcinogenesis. Although limited evidence is currently available, future studies may need to focus more on such dietary factors, especially for cancers in the digestive tract that have relatively high amounts of bioactive food components and PPARG expression.

## Figures and Tables

**Table 1:** Characteristics of studies included in the systematic review.

Cancer Site	Study Design *	First Author/Publication Year	Study Location	Age **	Race	Cases/Controls	Women (%)	Modifiable Factors	Case/CC	Case/CG	Case /GG	Control/CC	Control/CG	Control /GG
Colon/rectum	N CC	Vogel 2007 [29]	Denmark	59/56	NR	355/753	44%	Alcohol	252	96	7	550	190	13
Colon/rectum	PB CC	Murtaugh 2005 [23] and Slattery 2005 [24]	USA	30–79	NH White, Hispanic, Black, Asian, and Native American	1577/1971 (colon) 794/1001 (rectum)	45% (colon) 42% (rectum)	Dietary fats, energy, dietary antioxidants and food items ≠	1234 606	343 188		1493 790	478 211	
Colon/rectum	HB CC	Landi 2003 [26]	Spain	<58–>75	NR	377/326	43%	Vitamin A	311	46	3	243	61	5
Colon/rectum	HB CC	Jiang 2005 [25]	India	20–75	Asian	301/291	36%	Fish intake	240	57	4	230	57	4
Colon/rectum	HB CC	Kuriki 2006 [30]	Japan	57.9 (study 1) 58.9 (study 2)	NR	128/238 (study 1) 257/771 (study 2)	48% (study 1) 37% (study 2)	Meat, milk, and other food items ≠	120 248	7 9		221 732	17 37	
Colon/rectum	HB CC	Kim 2018 [31]	Korea	58.2	Asian	971/658	44%	Red meat intake	886	82	3	607	51	0
Prostate	N CC	Paltoo 2003 [28]	Finland	60.5	NR	193/188	0%	Dietary fat	121	64	8	128	54	6
Breast	N CC	Peterson 2012 [27]	Denmark	57/57	NR	798/798	100%	Alcohol	616	167	15	569	209	20

*: CC: Nested case-control study; HB CC: Hospital-based case-control study; PB CC: Population-based case-control study; BMI: Body mass index; NH: Non-Hispanic; NR: Not reported; **: Age is shown as mean/median among all participants, median among cases/controls [27,29], or the range among all participants [23,24,25,26].; ≠ Kuriki 2006 [30] included meat, milk, beef and pork, processed meat, poultry, fish, egg, milk, yogurt, mayonnaise, fried food, deep-fried food; Murtaugh 2005 [23] included dietary fats (total fat, saturated fat, monounsaturated fat, polyunsaturated fat, trans fat, cholesterol), dietary antioxidants (total tocopherol, β-Carotene, lutein, lycopene, vitamin C), foods (vegetables, refined grain, whole grain and Western and Prudent dietary patterns).

**Table 2:** Interactions between *PPARG2* Pro12Ala allele polymorphism and alcohol, fish, and fried food consumption in observational studies in relation to cancer risk.

						OR/IRR (95% CI)		
						*PPARG2* Pro12Ala Allele Polymorphism		
First Author/Publication Year	Age	Women (%)	Study Location	Cancer Site	Stratified Categories	CC	CG + GG	*p*-Value for Interaction
Petersen 2012 [27]	57/57	100%	Denmark	Breast cancer	Alcohol intake			
					Per 10 g alcohol/day	1.13 (1.04–1.23)	0.95 (0.83–1.08)	0.02
Vogel 2007 [29]	59/56	44%	Denmark	Colorectal cancer	Alcohol intake			
					Per 10 g alcohol/day	1.03 (0.96–1.10)	1.22 (1.07–1.39)	0.02
Jiang 2005 [25]	20–75	36%	India	Colorectal cancer	Fish intake			
					Low	Reference	1.14 (0.72–1.81)	0.36
					High	0.74 (0.46–1.18)	0.51 (0.20–1.27)	
Kuriki 2006 [30] (study 2)	58.9	37%	Japan	Colorectal cancer	Fish			
					Low	Reference	0.69 (0.23–2.10)	0.49
					Middle	0.93 (0.66–1.29)	1.09 (0.34–3.47)	
					High	0.91 (0.61–1.34)	0.34 (0.04–2.81)	
					Fried foods			
					Low	Reference	0.23 (0.03–1.82)	0.01
					Middle	0.94 (0.65–1.34)	0.20 (0.03–1.56)	
					High	0.77 (0.51–1.16)	2.04 (0.74–5.63)	
					Deep-fried foods			
					Low	Reference	0.45 (0.10–2.05)	0.17
					Middle	0.81 (0.59–1.13)	0.59 (0.19–1.77)	
					High	0.68 (0.44–1.03)	1.33 (0.32–5.49)	

CC will be translated into proline and GG will be translated into alanine; age is shown as mean/median among all participants [30], median among cases/controls [27,29], or the range among all participants [25].

**Table 3:** Interactions between *PPARG2* Pro12Ala allele polymorphism and antioxidant intake in observational studies in relation to cancer risk.

					OR (95% CI)			
					*PPARG2* Pro12Ala Allele Polymorphism			
First Author/Publication Year	Age (Range)	Women (%)	Study Location	Stratified Categories	CC	CG + GG	CC	CG + GG
Landi 2003 [26]	<58–>75	43%	Spain		Colorectal Cancer			
				Vitamin A intake				
				Low	Reference	0.25 (0.12–0.53)		
				High	Reference	1.08 (0.53–2.20) *		
Murtaugh 2005 [23]	30–79	45% (colon)	USA		Colon Cancer		Rectal Cancer	
		42% (rectum)		Beta-Carotene				
				High	Reference	0.71 (0.52–0.96)	Reference	1.08 (0.73–1.61)
				Middle	0.94 (0.77–1.16)	0.85 (0.65–1.13)	1.03 (0.77–1.37)	0.43 (0.94–2.18)
				Low	0.82 (0.65–1.02)	0.82 (0.61–1.10)	1.15 (0.83–1.58)	1.28 (0.83–1.97)
				Lutein				
				High	Reference	0.63 (0.44–0.89)	Reference	1.06 (0.72–1.57)
				Middle	0.89 (0.72–1.10)	0.82 (0.61–1.10)	0.95 (0.72–1.27)	1.19 (0.79–1.79)
				Low	0.90 (0.71–1.13)	0.90 (0.71–1.13) *	0.90 (0.64–1.25)	1.15 (0.73–1.79)
				Lycopene				
				High	Reference	0.75 (0.52–1.06)	Reference	0.89 (0.61–1.30)
				Middle	1.02 (0.82–1.27)	0.96 (0.72–1.29)	0.81 (0.61–1.08)	1.27 (0.85–1.92)
				Low	1.00 (0.80–1.26)	0.89 (0.67–1.19)	1.10 (0.81–1.48)	1.37 (0.89–2.09)
				Vitamin C				
				High	Reference	0.79 (0.58–1.06)	Reference	1.33 (0.90–1.97)
				Middle	1.05 (0.86–1.28)	0.90 (0.68–1.19)	1.19 (0.89–1.59)	1.31 (0.87–1.98)
				Low	0.88 (0.70–1.10)	0.86 (0.64–1.15)	1.28 (0.92–1.77)	1.46 (0.94–2.27)
				Total tocopherol				
				High	Reference	0.96 (0.71–1.30)	Reference	1.02 (0.72–1.42)
				Middle	1.02 (0.83–1.26)	0.85 (0.63–1.15)	0.96 (0.71–1.29)	1.36 (0.92–2.03)
				Low	0.96 (0.75–1.23)	0.82 (0.60–1.12)	1.21 (0.85–1.73)	1.55 (0.92–2.64)

CC will be translated into proline and GG will be translated into alanine. * star denotes *p*-value for interaction < 0.05.

**Table 4:** Interactions between *PPARG2* Pro12Ala allele polymorphism and diet patterns and vegetable intake in observational studies in relation to cancer risk.

					OR (95% CI)			
					*PPARG2* Pro12Ala Allele Polymorphism			
First Author/Publication Year	Age (Range)	Women (%)	Study Location	Stratified Categories	CC	CG + GG	CC	CG + GG
Murtaugh 2005 [23]	30–79	45% (colon) 42% (rectum)	USA		Colon Cancer		Rectal Cancer	
				Western dietary pattern				
				Low	Reference	0.71 (0.52–0.96)	Reference	1.40 (0.93–2.10)
				Middle	1.22 (1.00–1.49)	1.17 (0.89–1.54)	0.98 (0.74–1.30)	1.19 (0.79–1.79)
				High	1.27 (1.00–1.62)	1.18 (0.87–1.63)	1.13 (0.84–1.52)	1.17 (0.78–1.75)
				Prudent dietary pattern				
				High	Reference	0.66 (0.49–0.89)	Reference	1.29 (0.87–1.92)
				Middle	1.00 (0.82–1.23)	0.92 (0.69–1.22)	0.99 (0.74–1.34)	0.95 (0.61–1.47)
				Low	1.02 (0.81–1.28)	1.07 (0.79–1.45) *	1.07 (0.78–1.45)	1.36 (0.91–2.04)
				Vegetables				
				High	Reference	0.72 (0.54–0.96)	Reference	1.16 (0.79–1.71)
				Middle	0.91 (0.75–1.12)	0.81 (0.61–1.08)	0.95 (0.71–1.26)	0.93 (0.62–1.40)
				Low	0.94 (0.75–1.17)	0.96 (0.71–1.30)	0.87 (0.64–1.19)	1.28 (0.83–1.97)
				Refined grain				
				Low	Reference	0.70 (0.53–0.94)	Reference	1.68 (1.11–2.54)
				Middle	1.07 (0.88–1.29)	0.95 (0.72–1.24)	1.04 (0.75–1.43)	1.2 (0.78–1.59)
				High	1.08 (0.88–1.33)	1.17 (0.87–1.58) *	1.12 (0.83–1.51)	1.53 (1.03–2.28)
				Whole grain				
				High	Reference	0.74 (0.57–0.97)	Reference	1.24 (0.81–1.89)
				Middle	0.98 (0.81–1.19)	0.96 (0.73–1.28)	1.07 (0.81–1.43)	1.21 (0.79–1.84)
				Low	0.92 (0.74–1.13)	0.85 (0.63–1.15)	1.32 (0.93–1.87)	1.22 (0.78–1.92)

CC will be translated into proline and GG will be translated into alanine. * star denotes *p*-value for interaction < 0.05.

## Data Availability

Data sharing not applicable.

## Supplementary Materials

The following are available online at https://www.mdpi.com/2072-6643/13/1/261/s1, Supplementary method: List of search terms, Table S1: Risk of Bias Assessment, Table S2: Interactions between PPARG2 Pro12Ala polymorphism and energy, fat, or animal protein intakes in observational studies in relation to cancer risk.

## References

[1] Wang L., Waltenberger B., Pferschy-Wenzig E.M., Blunder M., Liu X., Malainer C., Blazevic T., Schwaiger S., Rollinger J.M., Heiss E.H. (2014). Natural product agonists of peroxisome proliferator-activated receptor gamma (PPARgamma): A review. Biochem. Pharmacol..

[2] Kroker A.J., Bruning J.B. (2015). Review of the Structural and Dynamic Mechanisms of PPARgamma Partial Agonism. PPAR Res..

[3] Michalik L., Desvergne B., Wahli W. (2004). Peroxisome-proliferator-activated receptors and cancers: Complex stories. Nat. Rev. Cancer.

[4] Peters J.M., Shah Y.M., Gonzalez F.J. (2012). The role of peroxisome proliferator-activated receptors in carcinogenesis and chemoprevention. Nat. Rev. Cancer.

[5] Janani C., Ranjitha Kumari B.D. (2015). PPAR gamma gene—A review. Diabetes Metab. Syndr..

[6] Vidal-Puig A.J., Considine R.V., Jimenez-Linan M., Werman A., Pories W.J., Caro J.F., Flier J.S. (1997). Peroxisome proliferator-activated receptor gene expression in human tissues. Effects of obesity, weight loss, and regulation by insulin and glucocorticoids. J. Clin. Investig..

[7] Penumetcha M., Santanam N. (2012). Nutraceuticals as Ligands of PPARgamma. PPAR Res..

[8] Tyagi S., Gupta P., Saini A.S., Kaushal C., Sharma S. (2011). The peroxisome proliferator-activated receptor: A family of nuclear receptors role in various diseases. J. Adv. Pharm. Technol. Res..

[9] Rizos C.V., Elisaf M.S., Mikhailidis D.P., Liberopoulos E.N. (2009). How safe is the use of thiazolidinediones in clinical practice?. Expert Opin. Drug Saf..

[10] Ortuno Sahagun D., Marquez-Aguirre A.L., Quintero-Fabian S., Lopez-Roa R.I., Rojas-Mayorquin A.E. (2012). Modulation of PPAR-gamma by Nutraceutics as Complementary Treatment for Obesity-Related Disorders and Inflammatory Diseases. PPAR Res..

[11] Willson T.M., Lambert M.H., Kliewer S.A. (2001). Peroxisome proliferator-activated receptor gamma and metabolic disease. Annu. Rev. Biochem..

[12] Robbins G.T., Nie D. (2012). PPAR gamma, bioactive lipids, and cancer progression. Front. Biosci..

[13] Stumvoll M., Haring H. (2002). The peroxisome proliferator-activated receptor-gamma2 Pro12Ala polymorphism. Diabetes.

[14] Wang W., Shao Y., Tang S., Cheng X., Lian H., Qin C. (2015). Peroxisome proliferator-activated receptor-gamma (PPARgamma) Pro12Ala polymorphism and colorectal cancer (CRC) risk. Int. J. Clin. Exp. Med..

[15] Tang W., Chen Y., Wang Y., Gu H., Chen S., Kang M. (2015). Peroxisome proliferator-activated receptor gamma (PPARG) polymorphisms and breast cancer susceptibility: A meta-analysis. Int. J. Clin. Exp. Med..

[16] Mansoori A., Amini M., Kolahdooz F., Seyedrezazadeh E. (2015). Obesity and Pro12Ala Polymorphism of Peroxisome Proliferator-Activated Receptor-Gamma Gene in Healthy Adults: A Systematic Review and Meta-Analysis. Ann. Nutr. Metab..

[17] Zhao J., Zhi Z., Song G., Wang J., Wang C., Ma H., Yu X., Sui A., Zhang H. (2015). Peroxisome proliferator-activated receptor-gamma Pro12Ala polymorphism could be a risk factor for gastric cancer. Asian Pac. J. Cancer Prev..

[18] Ruiz-Narvaez E.A., Kraft P., Campos H. (2007). Ala12 variant of the peroxisome proliferator-activated receptor-gamma gene (PPARG) is associated with higher polyunsaturated fat in adipose tissue and attenuates the protective effect of polyunsaturated fat intake on the risk of myocardial infarction. Am. J. Clin. Nutr..

[19] Memisoglu A., Hu F.B., Hankinson S.E., Manson J.E., De Vivo I., Willett W.C., Hunter D.J. (2003). Interaction between a peroxisome proliferator-activated receptor gamma gene polymorphism and dietary fat intake in relation to body mass. Hum. Mol. Genet..

[20] Zeggini E., Weedon M.N., Lindgren C.M., Frayling T.M., Elliott K.S., Lango H., Timpson N.J., Perry J.R., Rayner N.W., Freathy R.M. (2007). Replication of genome-wide association signals in UK samples reveals risk loci for type 2 diabetes. Science.

[21] Moher D., Liberati A., Tetzlaff J., Altman D.G., Group P. (2009). Preferred reporting items for systematic reviews and meta-analyses: The PRISMA statement. PLoS Med..

[22] Wells G.S.B., O’Connell D., Peterson J., Welch V., Losos M., Tugwell P. The Newcastle-Ottawa Scale (NOS) for Assessing the Quality of Nonrandomised Studies in Meta-Analyses. http://www.ohri.ca/programs/clinical_epidemiology/oxford.asp.

[23] Murtaugh M.A., Ma K.N., Caan B.J., Sweeney C., Wolff R., Samowitz W.S., Potter J.D., Slattery M.L. (2005). Interactions of peroxisome proliferator-activated receptor {gamma} and diet in etiology of colorectal cancer. Cancer Epidemiol. Biomark. Prev..

[24] Slattery M.L., Murtaugh M.A., Sweeney C., Ma K.N., Potter J.D., Caan B.J., Samowitz W. (2005). PPARgamma, energy balance, and associations with colon and rectal cancer. Nutr. Cancer.

[25] Jiang J., Gajalakshmi V., Wang J., Kuriki K., Suzuki S., Nakamura S., Akasaka S., Ishikawa H., Tokudome S. (2005). Influence of the C161T but not Pro12Ala polymorphism in the peroxisome proliferator-activated receptor-gamma on colorectal cancer in an Indian population. Cancer Sci..

[26] Landi S., Moreno V., Gioia-Patricola L., Guino E., Navarro M., de Oca J., Capella G., Canzian F., Bellvitge Colorectal Cancer Study G. (2003). Association of common polymorphisms in inflammatory genes interleukin (IL)6, IL8, tumor necrosis factor alpha, NFKB1, and peroxisome proliferator-activated receptor gamma with colorectal cancer. Cancer Res..

[27] Petersen R.K., Larsen S.B., Jensen D.M., Christensen J., Olsen A., Loft S., Nellemann C., Overvad K., Kristiansen K., Tjonneland A. (2012). PPARgamma-PGC-1alpha activity is determinant of alcohol related breast cancer. Cancer Lett..

[28] Paltoo D., Woodson K., Taylor P., Albanes D., Virtamo J., Tangrea J. (2003). Pro12Ala polymorphism in the peroxisome proliferator-activated receptor-gamma (PPAR-gamma) gene and risk of prostate cancer among men in a large cancer prevention study. Cancer Lett..

[29] Vogel U., Christensen J., Dybdahl M., Friis S., Hansen R.D., Wallin H., Nexo B.A., Raaschou-Nielsen O., Andersen P.S., Overvad K. (2007). Prospective study of interaction between alcohol, NSAID use and polymorphisms in genes involved in the inflammatory response in relation to risk of colorectal cancer. Mutat. Res..

[30] Kuriki K., Hirose K., Matsuo K., Wakai K., Ito H., Kanemitsu Y., Hirai T., Kato T., Hamajima N., Takezaki T. (2006). Meat, milk, saturated fatty acids, the Pro12Ala and C161T polymorphisms of the PPARgamma gene and colorectal cancer risk in Japanese. Cancer Sci..

[31] Kim N.H., Seol J.E., Kim J., Lee B.H., Hwang D.Y., Jeong J., Lee H.J., Ahn Y.O., Kim D.H., Lee J.E. (2019). Red meat intake, CYP2E1 and PPARgamma polymorphisms, and colorectal cancer risk. Eur. J. Cancer Prev..

[32] Wang P., Wang Q., Yin Y., Yang Z., Li W., Liang D., Zhou P. (2015). Association between Peroxisome Proliferator-activated Receptor Gamma Gene Polymorphisms and Atherosclerotic Diseases: A Meta-analysis of Case-control Studies. J. Atheroscler. Thromb..

[33] Liang X., Fan X., Tan K., Zhang L., Jian L., Yu L. (2018). Peroxisome proliferators-activated receptor gamma polymorphisms and colorectal cancer risk. J. Cancer Res. Ther..

[34] Ryan-Harshman M., Aldoori W. (2007). Diet and colorectal cancer: Review of the evidence. Can. Fam. Physician.

[35] La Vecchia C., Ferraroni M., Mezzetti M., Enard L., Negri E., Franceschi S., Decarli A. (1996). Attributable risks for colorectal cancer in northern Italy. Int. J. Cancer.

[36] The Human Protein Altas Tissue Expression of PPARG- Summary—The Human Protein Atlas. https://www.proteinatlas.org/ENSG00000132170-PPARG/tissue.

[37] Uhlen M., Fagerberg L., Hallstrom B.M., Lindskog C., Oksvold P., Mardinoglu A., Sivertsson A., Kampf C., Sjostedt E., Asplund A. (2015). Proteomics. Tissue-based map of the human proteome. Science.

[38] Adachi M., Kurotani R., Morimura K., Shah Y., Sanford M., Madison B.B., Gumucio D.L., Marin H.E., Peters J.M., Young H.A. (2006). Peroxisome proliferator activated receptor gamma in colonic epithelial cells protects against experimental inflammatory bowel disease. Gut.

[39] Salgia M.M., Elix C.C., Pal S.K., Jones J.O. (2019). Different roles of peroxisome proliferator-activated receptor gamma isoforms in prostate cancer. Am. J. Clin. Exp. Urol..

[40] Discacciati A., Orsini N., Wolk A. (2012). Body mass index and incidence of localized and advanced prostate cancer—A dose-response meta-analysis of prospective studies. Ann. Oncol..

[41] Nakles R.E., Kallakury B.V., Furth P.A. (2013). The PPARgamma agonist efatutazone increases the spectrum of well-differentiated mammary cancer subtypes initiated by loss of full-length BRCA1 in association with TP53 haploinsufficiency. Am. J. Pathol..

[42] Dong J.T. (2013). Anticancer activities of PPARgamma in breast cancer are context-dependent. Am. J. Pathol..

[43] Zaytseva Y.Y., Wallis N.K., Southard R.C., Kilgore M.W. (2011). The PPARgamma antagonist T0070907 suppresses breast cancer cell proliferation and motility via both PPARgamma-dependent and -independent mechanisms. Anticancer. Res..

[44] Jiang W.G., Douglas-Jones A., Mansel R.E. (2003). Expression of peroxisome-proliferator activated receptor-gamma (PPARgamma) and the PPARgamma co-activator, PGC-1, in human breast cancer correlates with clinical outcomes. Int. J. Cancer.

