# Supplementary material for: Diet and PPARG2 Pro12Ala Polymorphism Interactions in Relation to Cancer Risk: A Systematic Review
Source: Nutrients. 2021 Jan 18;13(1):261. doi: 10.3390/nu13010261 (PMC7831057; doi:10.3390/nu13010261)
Supplement: Supplementary file 1 [file nutrients-13-00261-s001.pdf]

Supplementary materials

Methods

List of search terms:

("Peroxisome proliferator-activated receptor"[All Fields] OR "Peroxisome proliferator activated receptor"[All Fields] OR "Peroxisome proliferator-activated receptors"[All Fields] OR "Peroxisome proliferator activated receptors"[All Fields] OR ("peroxisome proliferator-activated receptors"[MeSH Terms] OR ("peroxisome"[All Fields] AND "proliferator-activated"[All Fields] AND "receptors"[All Fields]) OR "peroxisome proliferator-activated receptors"[All Fields] OR "ppar"[All Fields])) AND (("neoplasms"[MeSH Terms] OR "neoplasms"[All Fields] OR "cancer"[All Fields]) OR (("carcinoma"[MeSH Terms] OR "carcinoma"[All Fields]) OR carcinoma'[All Fields] OR carcinoma''[All Fields] OR carcinoma's[All Fields] OR carcinoma,[All Fields] OR carcinoma26[All Fields] OR carcinomaa[All Fields] OR carcinomaand[All Fields] OR carcinomaarising[All Fields] OR carcinomaassociated[All Fields] OR carcinomaban[All Fields] OR carcinomabearing[All Fields] OR carcinomabol[All Fields] OR carcinomac[All Fields] OR carcinomacekwxwith[All Fields] OR carcinomacell[All Fields] OR carcinomacells[All Fields] OR carcinomacomparative[All Fields] OR carcinomadagger[All Fields] OR carcinomade[All Fields] OR carcinomaderived[All Fields] OR carcinomadeveloped[All Fields] OR carcinomadouble[All Fields] OR carcinomae[All Fields] OR carcinomaendometriale[All Fields] OR carcinomaepidermal[All Fields] OR carcinomagenesis[All Fields] OR carcinomahad[All Fields] OR carcinomahcc[All Fields] OR carcinomahep[All Fields] OR carcinomahnliche[All Fields] OR carcinomahoz[All Fields] OR carcinomai[All Fields] OR carcinomain[All Fields] OR carcinomainak[All Fields] OR carcinomais[All Fields] OR carcinomaja[All Fields] OR carcinomajaban[All Fields] OR carcinomajabol[All Fields] OR carcinomajanak[All Fields] OR carcinomak[All Fields] OR carcinomakban[All Fields] OR carcinomakrol[All Fields] OR carcinomal[All Fields] OR carcinomalarda[All Fields] OR carcinomalari[All Fields] OR carcinomalike[All Fields] OR carcinomametastasis[All Fields] OR carcinoman[All Fields] OR carcinomanak[All Fields] OR carcinomans[All Fields] OR carcinomaoesophageal[All Fields] OR carcinomaof[All Fields] OR carcinomaon[All Fields] OR carcinomaoral[All Fields] OR carcinomaosccand[All Fields] OR carcinomaoverview[All Fields] OR carcinomapatients[All Fields] OR carcinomaporo[All Fields] OR carcinomaprostatic[All Fields] OR carcinomara[All Fields] OR carcinomarol[All Fields] OR carcinomars[All Fields] OR ("carcinoma"[MeSH Terms] OR "carcinoma"[All Fields] OR "carcinomas"[All Fields]) OR carcinomas'[All Fields] OR carcinomas'is[All Fields] OR carcinomasa[All Fields] OR carcinomasand[All Fields] OR carcinomasarcoma[All Fields] OR carcinomasas[All Fields] OR carcinomasitu[All Fields] OR carcinomasl[All Fields] OR carcinomastenose[All Fields] OR carcinomastosis[All Fields] OR carcinomaswith[All Fields] OR carcinomasymptoms[All Fields] OR carcinomasyndrome[All Fields] OR carcinomat[All Fields] OR carcinomata[All Fields] OR carcinomatas[All Fields] OR carcinomatasis[All Fields] OR carcinomate[All Fields] OR carcinomateous[All Fields] OR carcinomateuse[All Fields] OR carcinomateuses[All Fields] OR carcinomateux[All Fields] OR carcinomateuze[All Fields] OR carcinomathrombus[All Fields] OR carcinomatic[All Fields] OR carcinomatis[All Fields] OR carcinomatissue[All Fields]

OR carcinomato[All Fields] OR carcinomatoasa[All Fields] OR carcinomatoase[All Fields] OR carcinomatodes[All Fields] OR carcinomatoese[All Fields] OR carcinomatoesen[All Fields] OR carcinomatoeser[All Fields] OR carcinomatoid[All Fields] OR carcinomatoides[All Fields] OR carcinomatoids[All Fields] OR carcinomatos[All Fields] OR carcinomatosa[All Fields] OR carcinomatosae[All Fields] OR carcinomatosas[All Fields] OR carcinomatosat[All Fields] OR carcinomatose[All Fields] OR carcinomatosem[All Fields] OR carcinomatosen[All Fields] OR carcinomatoser[All Fields] OR ("carcinoma"[MeSH Terms] OR "carcinoma"[All Fields] OR "carcinomatoses"[All Fields]) OR carcinomatosi[All Fields] OR carcinomatosia[All Fields] OR ("carcinoma"[MeSH Terms] OR "carcinoma"[All Fields] OR "carcinomatosis"[All Fields]) OR carcinomatosis'[All Fields] OR carcinomatosis''[All Fields] OR carcinomatosisapropos[All Fields] OR carcinomatosist[All Fields] OR carcinomatoso[All Fields] OR carcinomatosos[All Fields] OR carcinomatosum[All Fields] OR carcinomatosus[All Fields] OR carcinomatous[All Fields] OR carcinomatousa[All Fields] OR carcinomatousis[All Fields] OR carcinomatously[All Fields] OR carcinomatoza[All Fields] OR carcinomatoze[All Fields] OR carcinomatozei[All Fields] OR carcinomatozis[All Fields] OR carcinomatrichoscopy[All Fields] OR carcinomatus[All Fields] OR carcinomatusum[All Fields] OR carcinomaval[All Fields] OR carcinomawas[All Fields] OR carcinomawere[All Fields] OR carcinomaxanti[All Fields])) AND ("humans"[MeSH Terms] AND English[lang])

Table S1. Risk of Bias Assessment

First author publication year	Selection↑	ComparabilityΔ	Outcome≺	Total
Petersen 2012 [27]	****	**	***	9
Kuriki 2006 [30]	***	**	***	8
Jiang 2005 [25]	***	**	***	8
Landi 2003 [26]	****	**	**	8
Paltoo 2003 [28]	****	**	**	8
Murtaugh 2005 [23] and Slattery 2005 [24]	***	**	***	8
Vogel 2007 [29]	****	**	***	9
Kim 2018 [31]	***	**	***	9

↑A maximum of 5 stars could be awarded for this item.

Δ A maximum of 2 stars could be awarded for this item.

≺A maximum of 3 stars could be awarded for this item.

Table S2. Interactions between *PPARG2* Pro12Ala allele polymorphism and energy, fat, or animal protein intakes in observational studies in relation to cancer risk

						OR (95% CI)		
						PPARG2 Pro12Ala allele polymorphism		
**First author/ publication year**	**Age (mean or range)**	**Women (%)**	**Study location**	**Cancer site**	**Stratified categories**	**CC**	**CG+GG**	***P*-value for Interaction**
Slattery 2005 [24]	30-79	45%	USA	Colon cancer	Energy intake			
					Low	Reference	0.8 (0.6-1.1)	0.89
					Middle	1.3 (1.0-1.5)	1.1 (0.8-1.4)	
					High	1.6 (1.3-2.1)	1.6 (1.1-2.2)	
Slattery 2005 [24]	30-79	42%	USA	Rectal cancer	Energy intake			
					Low	Reference	1.2 (0.8-1.9)	0.17
					Middle	1.0 (0.7-1.3)	1.6 (1.1-2.4)	
					High	1.3 (0.9-1.8)	1.2 (0.8-1.8)	
Paltoo 2003 [28]	60.5	0%	Finland	Prostate cancer	Dietary fat	NR	NR	>0.05
Kim 2018 [31]	58.2	44%	Korea	Colorectal cancer	Red Meat intake			
					Low	Reference	Reference	
					Low middle	1.44 (1.00-2.08)	0.48 (0.13-1.75)	0.52
					High middle	1.21 (0.81-1.80)	0.70 (0.17-2.94)	
					High	1.47 (0.92-2.35)	0.49 (0.11-2.22)	
Kuriki 2006 [30]	57.9 (study 1) 58.9 (study 2)	48% (study 1) 37% (study 2)	Japan	Colorectal cancer	Meat (study 1)			
					Low	Reference	0.56 (0.05-6.24)	0.64
					Middle	1.94 (1.06-3.58)	0.93 (0.26-3.34)	
					High	2.01 (0.91-4.45)	2.48 (0.27-22.82)	
					Milk (study 1)			
					Low	Reference	1.47 (0.11-19.47)	0.08
					Middle	0.61 (0.32-1.16)	1.08 (0.25-4.75)	
					High	0.63 (0.34-1.16)	0.08 (0.01-0.69)	
					Beef and pork (study 2)			
					Low	Reference	0.32 (0.04-2.60)	0.55
					Middle	1.07 (0.75-1.53)	1.06 (0.37-3.00)	
					High	1.04 (0.69-1.58)	0.89 (0.24-3.32)	
					Processed meat (study 2)			

					Low	Reference	0.65 (0.19-2.28)	0.81
					Middle	1.34 (0.96-1.88)	1.36 (0.47-3.90)	
					High	1.09 (0.71-1.67)	0.43 (0.05-3.51)	
					Poultry (study 2)			
					Low	Reference	0.29 (0.04-2.28)	0.25
					Middle	0.79 (0.57-1.09)	0.88 (0.31-2.49)	
					High	0.78 (0.49-1.23)	0.83 (0.22-3.07)	
					Egg (study 2)			
					Low	Reference	0.41 (0.09-1.85)	0.07
					Middle	0.77 (0.55-1.08)	0.15 (0.02-1.12)	
					High	0.75 (0.50-1.11)	2.15 (0.64-7.22)	
					Milk (study 2)			
					Low	Reference	1.31 (0.48-3.55)	0.39
					Middle	0.98 (0.69-1.40)	0.22 (0.03-1.74)	
					High	0.67 (0.46-0.97)	0.48 (0.10-2.23)	
					Yoghurt (study 2)			
					Low	Reference	0.74 (0.28-2.00)	0.70
					Middle	1.40 (1.00-1.95)	0.90 (0.19-4.31)	
					High	1.14 (0.73-1.77)	1.45 (0.27-7.67)	
					Mayonnaise (study 2)			
					Low	Reference	0.47 (0.10-2.12)	0.62
					Middle	0.84 (0.59-1.18)	1.02 (0.32-3.23)	
					High	1.12 (0.76-1.64)	0.90 (0.24-3.34)	
Murtaugh 2005 [23]	30-79	45%	USA	Colon cancer	Total fat			
					Low	Reference	0.93 (0.71-1.22)	NR
					Middle	1.18 (0.97-1.43)	0.88 (0.66-1.17)	
					High	1.13 (0.87-1.48)	1.09 (0.78-1.53)	
					Saturated fat			
					Low	Reference	0.89 (0.67-1.17)	NR
					Middle	1.01 (0.85-1.25)	0.81 (0.61-1.08)	
					High	1.09 (0.84-1.41)	1.01 (0.73-1.40)	
					Monounsaturated fat			
					Low	Reference	0.91 (0.70-1.20)	NR
					Middle	1.23 (1.01-1.50)	0.90 (0.67-1.20)	
					High	1.02 (0.78-1.33)	1.04 (0.74-1.46)	
					Polyunsaturated fat			
					Low	Reference	0.83 (0.64-1.08)	NR
					Middle	1.10 (0.91-1.32)	1.11 (0.84-1.45)	
					High	1.13 (0.87-1.46)	0.88 (0.62-1.25)	

					Trans fat			
					Low	Reference	0.85 (0.63-1.12)	NR
					Middle	1.20 (0.99-1.47)	0.88 (0.65-1.17)	
					High	1.24 (0.98-1.56)	1.31 (0.97-1.77)	
					Cholesterol			
					Low	Reference	0.86 (0.65-1.14)	NR
					Middle	1.16 (0.95-1.42)	0.97 (0.73-1.29)	
					High	1.25 (0.99-1.59)	1.17 (0.86-1.61)	
Murtaugh 2005 [23]	30-79	42%	USA	Rectal cancer	Total fat			
					Low	Reference	1.41 (0.89-2.25)	NR
					Middle	0.92 (0.68-1.24)	1.24 (0.80-1.91)	
					High	1.02 (0.72-1.45)	1.03 (0.68-1.57)	
					Saturated fat			
					Low	Reference	1.19 (0.79-1.84)	NR
					Middle	0.93 (0.70-1.24)	1.16 (0.75-1.77)	
					High	1.03 (0.72-1.46)	1.17 (0.78-1.80)	
					Monounsaturated fat			
					Low	Reference	1.78 (1.10-2.88)	NR
					Middle	1.05 (0.80-1.42)	1.04 (0.67-1.62)	
					High	1.03 (0.72-1.47)	1.14 (0.75-1.73)	
					Polyunsaturated fat			
					Low	Reference	1.31 (0.76-2.25)	NR
					Middle	0.98 (0.71-1.35)	1.22 (0.77-1.93)	
					High	0.87 (0.62-1.23)	0.99 (0.66-1.48)	
					Trans fat			
					Low	Reference	1.70 (1.11-2.59)	NR
					Middle	1.04 (0.79-1.38)	1.03 (0.69-1.55)	
					High	1.03 (0.73-1.44)	1.10 (0.72-1.68)	
					Cholesterol			
					Low	Reference	1.12 (0.72-1.75)	NR
					Middle	1.03 (0.76-1.38)	1.66 (1.10-2.50)	
					High	1.54 (1.12-2.12)	1.52 (1.00-2.31)	

CC will be translated into proline and GG will be translated into alanine. NR: not reported
